# The Responses of Medical General Practitioners to Unreasonable Patient Demand for Antibiotics - A Study of Medical Ethics Using Immersive Virtual Reality

**DOI:** 10.1371/journal.pone.0146837

**Published:** 2016-02-18

**Authors:** Xueni Pan, Mel Slater, Alejandro Beacco, Xavi Navarro, Anna I. Bellido Rivas, David Swapp, Joanna Hale, Paul Alexander George Forbes, Catrina Denvir, Antonia F. de C. Hamilton, Sylvie Delacroix

**Affiliations:** 1 Institute of Cognitive Neuroscience, University College London, London, United Kingdom; 2 Department of Computing, Goldsmiths College, University of London, London, United Kingdom; 3 Event Lab, Faculty of Psychology, University of Barcelona, Barcelona, Spain; 4 Institució Catalana de Recerca i Estudis Avançats (ICREA), Barcelona, Spain; 5 Department of Computer Science, University College London, London, United Kingdom; 6 UCL Faculty of Laws, University College London, London, United Kingdom; University of South Australia, AUSTRALIA

## Abstract

**Background:**

Dealing with insistent patient demand for antibiotics is an all too common part of a General Practitioner’s daily routine. This study explores the extent to which portable Immersive Virtual Reality technology can help us gain an accurate understanding of the factors that influence a doctor’s response to the ethical challenge underlying such tenacious requests for antibiotics (given the threat posed by growing anti-bacterial resistance worldwide). It also considers the potential of such technology to train doctors to face such dilemmas.

**Experiment:**

Twelve experienced GPs and nine trainees were confronted with an increasingly angry demand by a woman to prescribe antibiotics to her mother in the face of inconclusive evidence that such antibiotic prescription is necessary. The daughter and mother were virtual characters displayed in immersive virtual reality. The specific purposes of the study were twofold: first, whether experienced GPs would be more resistant to patient demands than the trainees, and second, to investigate whether medical doctors would take the virtual situation seriously.

**Results:**

Eight out of the 9 trainees prescribed the antibiotics, whereas 7 out of the 12 GPs did so. On the basis of a Bayesian analysis, these results yield reasonable statistical evidence in favor of the notion that experienced GPs are more likely to withstand the pressure to prescribe antibiotics than trainee doctors, thus answering our first question positively. As for the second question, a post experience questionnaire assessing the participants’ level of presence (together with participants’ feedback and body language) suggested that overall participants did tend towards the illusion of being in the consultation room depicted in the virtual reality and that the virtual consultation taking place was really happening.

## Introduction

Despite widely reported campaigns aimed at raising awareness of the “slowly emerging disaster” [1] inherent in growing antimicrobial resistance, there is evidence of continuing over-prescription of antibiotics [2]. The WHO cites ‘inappropriate and irrational use of medicines’ as a major contributor to the rise of antibiotic-resistant infections [3]. Among the strategies aimed at curbing antibiotic resistance, control of “inappropriate” use by frontline doctors is prevalent. While there is some debate about the efficiency of particular prescribing restriction strategies [4], it is clear that if we are to avert (or at any rate mitigate) the very likely prospect of a “post-antibiotic age” we must “move some way from the patterns of antibiotic access and use that we currently tolerate” [5]. It is also clear that rigorous microbial stewardship demands careful (re)consideration of the relationship between patient autonomy and justice [6–9].

Deploring the fact that, when balancing the risk of suboptimal treatment against the public interest in controlling antibiotic resistance, “many physicians are reluctant to impose even small avoidable risks on patients”, Michael Millar convincingly argued that antibiotics should only be used “to ameliorate some substantial risk of irretrievable harm” [5]. In contrast, a recent UK report [10] suggested that UK doctors make 10 million prescriptions per year that are not justified by (even modest) clinical needs.

In fact, it is known that doctors are influenced by many factors when deciding to prescribe, and that many of these factors are neither clinically nor ethically relevant. Top amongst these is the otherwise laudable aim of pleasing the patient. A recent cross-sectional study using practice-level data from UK primary care databases reported that antibiotic prescribing volume is a “significant positive predictor of all ‘doctor satisfaction’ and ‘practice satisfaction’ scores in the General Practice Patient Survey, and was the strongest predictor of overall satisfaction out of 13 prescribing variables” [11]. This does not bode well for antibiotic stewardship, given the impact of those satisfaction scores on GPs’ pay-for-performance Quality and Outcomes framework. Similarly, Bradley [12] found that social factors in the relationship between doctors and patient play an overly large role in prescribing. Amongst 70 doctors surveyed 70% mentioned the prescription of antibiotics as causing them the most discomfort—the highest percentage by far amongst all drugs mentioned, the next being benzodiazepines mentioned by 44%, followed by cardiovascular mentioned by 26%. In prescriptions for children, Mangione-Smith et al. [13] found that perceived parental expectations played a major role in the prescription of antibiotics for viral infections, and a later study found that parental misconceptions about the utility of antibiotics was a prevalent factor, especially amongst parents relying on Medicaid in the United States, as reported by Kleinman et al. [14], compared to parents relying on private medical insurance. In a systematic survey of past research, although reporting a wide heterogeneity in the methodology and results of studies, Lopez-Vazquez et al. [15] found that one important factor in the prescription of antibiotics was doctors’ fears about outcomes for their patients were antibiotics not to be prescribed.

The vast majority of work in this area relies on self-report by patients and doctors, or in-depth interviews of doctors—e.g. by Mattick et al. [16]—there being little opportunity to systematically study the prescription behavior of doctors under controlled laboratory conditions. In this paper we show how we have used immersive virtual reality (IVR) to confront doctors with the ethical challenge involved in dealing with strong patient demand for antibiotics in the face of inconclusive evidence that such antibiotic prescription is necessary (the patient is over 65 and presenting with a cough, so that there is only a small risk that not prescribing may cause preventable harm).

It has been pointed out by Blascovich et al. [17] that IVR provides a useful tool for the study of social behavior, and it has been argued to be especially useful in circumstances where for practical or ethical reasons ecologically valid studies are not feasible using human actors [18]. Indeed one of the most controversial ever social psychology studies, Stanley Milgram’s obedience to authority experiments—summarized in his 1974 book [19]—was partially replicated using IVR where the participants (‘Teachers’) were required to give electric shocks to an entirely virtual character (the ‘Learner’) when it gave incorrect answers to questions [20]. IVR has also been used to study bystander reactions to a violent incident [21], and in ethical dilemmas [22, 23].

We carried out an exploratory study with medical General Practitioners. The GPs who were either trainees or experienced, were faced with a very strong and increasingly angry demand to prescribe antibiotics to a patient despite the fact that her symptoms mostly suggested a viral rather than bacterial infection. Our ultimate goal is to consider whether such techniques could be routinely used in medical training. The specific purposes of the study were twofold: first, to investigate whether medical doctors would take the virtual situation seriously, and second, whether GPs with longer experience would be more resistant to patient demands than the more junior trainee doctors.

## Methods

### Ethics Statement

The study was approved by and carried out in accordance with the regulations of the Research Ethics Committee of UCL. Participants gave written informed consent on a form devised for this purpose that had been approved by the said Research Ethics Committee. The individual in the figure of this manuscript, and the individuals in the video, have given written informed consent (as outlined in PLOS consent form) to publish these case details.

### Scenario Details

**Fig 1:**
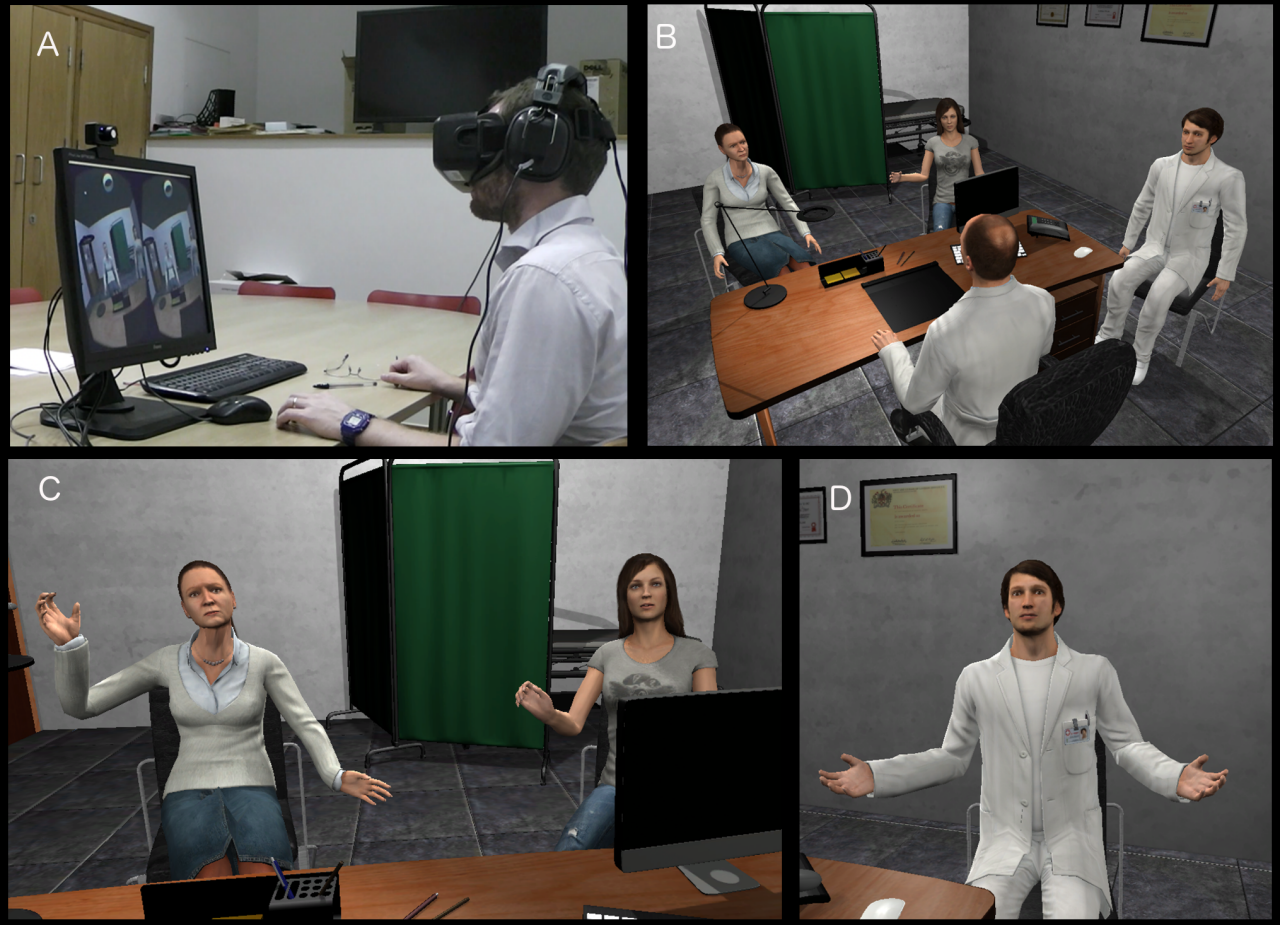
The physical setup and virtual scenario. (A) The participant wearing the head-mounted display and seated at the real desk that was registered with the virtual desk in virtual reality. (B) An overview of the scenario—the participant occupied the doctor position behind the desk. The medical student was seated to the right of the participant and the patient on the other side of the desk to the left, with her daughter to the right. (C) The patient and her daughter in conversation with the participant (D) the medical student.

A virtual consultation room (Fig 1) was created in Autodesk 3DsMax. It was modeled to look like a typical GP consultation in the United Kingdom—with a desk in front of the GP, a few chairs behind the desk, a bed in the far right corner of the room with a green panel screen room divider, and a door located on the far left corner of the room. On the desk in front of the GP there were a few stationary items, and a desktop monitor.

If the participants looked down, they would see a virtual body, substituting their own, dressed in healthcare white coat, with both their hands resting on the desk. A male virtual character sat to their right, also wearing the healthcare white coat, who introduced himself as “David Portillo, a medical student” (Fig 1D). David initiated a friendly conversation with the participant until there was a buzz sound, indicating that the next patient had arrived.

After the door opened, two virtual characters entered the room—representing an older lady (Ms Garcia) and her daughter (Elena) and they sat in front of the participant (Fig 1C). Elena started the conversation by complaining about the time that they had had to wait. When asked the reason for visiting the Doctor, Ms Garcia explained that she had a sore throat for a couple of days, and had developed a mild cough that morning. The participant could then have a discussion with Ms Garcia, who replied to some of the questions with a clear “Yes” or “No”, and others with a more vague answer, for instance: “I don’t remember”, or “Sometimes”. When the participant asked about her medical indicators the medical student, David, switched on the computer and read out the test results for Ms Garcia, which were all within the range of normal measurement (BP: 115/75 mm Hg; temperature: 36.5°C; pulse: 72 BPM; throat a bit red and sore, no ear infection). Overall, her symptoms suggested that Ms Garcia’s sore throat and cough were likely to be caused by a viral infection.

At this point the daughter, Elena, suggested: “My mother had exactly the same thing last year. They gave her antibiotics and she immediately got better. So all she needs is some antibiotics this time.” If the participant disagreed, she then continued to press for antibiotics with a variety of different arguments. One argument was that she would be on holiday and leave her mother alone for the next couple of days, and that her mother would not be able to travel to see the GP on her own. If the participant still did not prescribe antibiotics, she argued angrily that “This is so unfair. It’s going to ruin our holiday. I’m going to take this up with the local health authority; I think you are unfairly denying medicine to my mother....”; and “They say on the news that old people are just invisible, not treated with respect by the NHS.”

The scenario lasted around 8 minutes. See S1 File and S1 Video for full descriptions of the scenario.

### Materials

The Virtual Reality 3D imagery was rendered from a desktop machine through an nVidia GTX970 to an Oculus Rift (Developer Kit 2), a lightweight head-mounted display (HMD), which delivers 3D graphics at a resolution of 1080 x 1200 per eye, and a frame-rate of 75 Hz. Participants also wore over-ear headphones. The VR application was programmed in Unity3D. The experimenter triggered events through a control panel (Unity client application), that ran on a separate laptop, which communicated directly via an Ethernet cable with the desktop machine (Unity server application). The reason for the direct Ethernet cable was to enable execution of the Unity Server-Client without an Internet connection. The laptop allowed the experimenter to see the control panel and to observe the experiment via the monitor on the desktop at the same time. There was also a pair of external speakers plugged to the desktop machine, which was only turned on after the experiment started. We also videotaped the experiment from behind the participants, as well as the desktop monitor (Fig 1).

We intentionally designed our equipment so that it could all fit in a mobile storage case giving the flexibility of running the experiment at different venues. This is important for the purpose of this study because (a) it allowed us to conduct the experiment, with those medical professionals who required this, at their workplace making it easier to recruit participants, and (b) being able to run the experiment at professional venues (in our case GP practices) could make the whole experience more feasible for ultimate training purposes.

### Procedures

Twenty-one participants attended our study, 12 GPs and 9 trainees. The majority of participants (18) visited the lab at ICN for the experiment. For the other three participants, the experimenters took the equipment to their practice and conducted the experiment in their office.

Participants were asked to complete a set of questionnaires before the study. These pre-questionnaires included demographic questions and personality scales (see the section ‘Response Variables’ below).

Each participant was seated at a desk in a quiet room and given safety information about the Oculus Rift Headsets. Each participant was told that if they felt uncomfortable during the virtual consultation, they should close their eyes and clearly say: “Stop.” No participants asked to stop during the consultation. Each participant was told they would be taking part in a virtual consultation, and that their next patient would be Mrs Garcia, a woman of 78, who was coming in with her daughter. They were asked to behave as if it were a real consultation.

Participants donned the Oculus headset and were asked to place their hands on the desk in front. The researcher calibrated the headset to ensure that participants saw a virtual body in place of their real body, and that their posture matched that of the virtual body. The participants were asked to turn their head to orient themselves in the consultation room and to make sure that they saw the medical student. Following the calibration the researcher placed headphones on the participants, checked that the sound was audible and then informed them that the consultation was about to start. The researcher then started the video recording.

At that point the researcher triggered the start of the consultation and followed the script flow (S1 Fig) by clicking buttons from the control panel, similar to method used in a previous paper by Pan et al. [24]. The consultation lasted between 6–12 minutes. The researcher terminated the consultation if the participant agreed to give a prescription of antibiotics. If the participant did not agree to give a prescription, the consultation was terminated at the end of the script flow. Upon termination the display went dark and the researcher informed the participant that the consultation was over.

Following the consultation, the participants were asked to view the video recording and provide a running commentary of their thoughts and feelings during the consultation. This commentary was also recorded. Finally, participants completed further questionnaires concerning their decisions during the consultation and the usefulness of IVR as a training tool. All participants were reimbursed for their time at the end of the experiment (£30 store vouchers).

### Response Variables

Prior to the experiment participants completed the 10-item NEO ‘big five’ personality inventory [25] covering Extraversion, Agreeableness, Conscientiousness, Neuroticism and Openness. They also completed the Oldenburg Burnout Inventory [26] which measures exhaustion and disengagement from work, and a 5-item Professional Identification Scale, which measures the extent to which individuals identify with their profession and their colleagues [27, 28].

Various measures were recorded online such as the proportion of time that the doctor looked towards the medical student (GazeToStudent), the mother (GazeToMother) and daughter (GazeToDaughter).

In order to partially address our first question, regarding how the doctors would respond to this situation being presented in IVR a post experience questionnaire assessed their level of presence [29]. This has been argued to have two separate dimensions—the extent to which there is the illusion of being in the scenario (Place Illusion, PI) and the extent to which there is the illusion that the events taking place were really happening (Plausibility, Psi) [30]. These were assessed using questionnaires that we have used several times before (e.g. [31, 32]). We measure Place Illusion by the mean of 5 questions, and Plausibility as the mean of 10 questions. Each response was on a Likert scale of 1 (no illusion) to 7 (strong illusion), and presented in full in S2 File.

The major response variable of the study was whether or not the doctors actually prescribed antibiotics by the end of the virtual encounter (variable Prescribed). This was assessed from their answer to the post questionnaire. Prescribed is a binary variable scored as 0 ‘did not prescribe’ or 1 ‘did prescribe’ the antibiotics.

We present the quantitative measurements with statistical analysis, and qualitative insights from participants’ comments related to (1) the extent to which the scenario leads to feelings/emotions similar to real-life experience; (2) the potential of using VR application in trainings for healthcare; (3) critical comments requiring improvements in the system and scenario.

### Statistical Method

The software used for the Bayesian method was the JAGS system [33], together with MATLAB using MATJAGS (psiexp.ss.uci.edu/research/programs_data/jags), and some graphs were produced using Stata 14. Note that all prior distributions on the model parameters were chosen to have very large variance. For the analysis in the Section ‘Prescription’ in Results the simulation was run with 7 chains each consisting of 20,000 samples with a burnin of 2000. For the more complex model (Section ‘Presence and Prescription’ in Results) 7 chains were used each with a sample size of 150,000, and a burnin of 3000. The Rhat values of all parameters were equal to 1.00 (i.e., to 2 decimal places) indicating good convergence and similarity across the chains.

## Results

### Participants

Twenty-one participants were recruited via email adverts to local GP surgeries and GP training mailing lists. All were medical doctors, 9 of whom were Trainees with mean ± SD 0.55 ± 1.13 years of post-qualification experience as a general practitioner with age range 25–40, and 12 were GPs with 6.17 ± 7.8 years of experience, with age range 25–60. All the trainees were female, as were 7 of the GPs. We use the term Experience to refer to the factor with two levels (Trainees, GPs). The graphs showing participants’ distributions of the personality inventory and attitudes to work of this by Experience are presented in S2–S4 Figs.

### Gaze

The proportions of time that the Trainees and GPs spent looking at these characters were similar (S3 File).

### Presence

**Fig 2:**
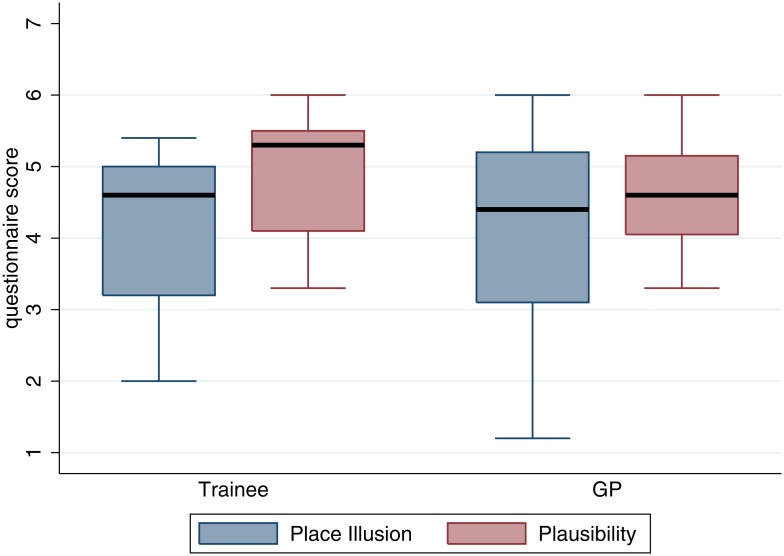
Box plot of Place Illusion and Plausibility by Experience. The thick horizontal black lines are the medians, and the boxes are the interquartile ranges.

S3 File shows the full set of results on all of the presence questionnaires. Here we concentrate on the average responses representing Place Illusion and Plausibility. Fig 2 shows the box plots for these two responses by Experience. Overall there are no differences between the GPs and Trainees, the medians are at least 4 (the mid-point of the scale), and in the case of Plausibility the bounds of the interquartile ranges are above 4. On the whole participants tended to have the illusion of being in the doctor surgery and tended to respond realistically.

### Prescription

**Fig 3:**
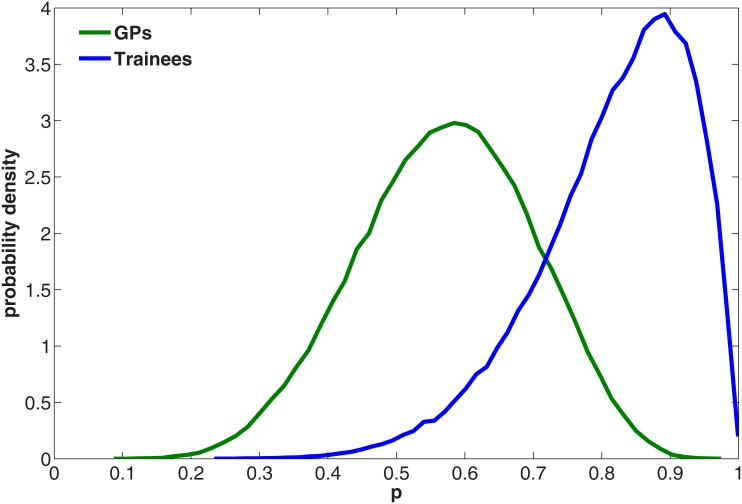
Posterior distributions of *p* by Experience, compared to the prior distribution that p has a uniform distribution on [0,1].

Full data is available in S4 File. Eight out of the 9 Trainees prescribed the antibiotics, whereas 7 out of the 12 GPs did so. If we let *p* be the probability of prescription with a prior uniform distribution on [0,1], and consider the number of doctors that prescribed the antibiotics as a binomially distributed random variable with probability *p*, then a simple Bayesian analysis results in the posterior distributions for *p* as shown in Fig 3.

From these distributions we can obtain posterior probabilities illustrating the difference between the two conditions. For example, for the Trainees the posterior probability that *p* is at least 0.75 is 0.08, whereas for the GPs it is 0.76. Similarly the probability that *p* is at least 0.9 is 0.0008 for the Trainees and 0.26 for the GPs. The probability that *p* for the GPs is greater than *p* for the Trainees is 0.92. Although certainly not overwhelming the statistical model provides evidence in favor of the notion that GPs are less likely to prescribe the antibiotics than the Trainees.

### Presence and Prescription

Here we put together our two questions and consider whether the extent of presence (Place Illusion and Plausibility) might influence the tendency to prescribe the antibiotics. This is worth considering because greater illusions of presence are argued to correspond with behavior that is more likely to match behavior in equivalent real world situations [29]. In order to address this question we consider the following statistical model. Let *p*_*i*_ be the probability that the *i*th doctor would prescribe (*i* = 1,2,…,n; *n* = 31). We then apply a standard binary logistic regression where:

$$\text{log}\left(\frac{p_{i}}{1-p_{i}}\right)=\beta_{0}+\beta_{1}G_{i}+\beta_{2}\Pi_{i}+\beta_{3}G_{i}\Pi_{i}+\beta_{4}\Psi_{i}+\beta_{5}G_{i}\Psi_{i}\text{K}$$ (1)

and *G*_*i*_ = 1 for GP and 0 for Trainee, Π_*i*_ is the Place Illusion score for the *i*th doctor, and Ψ_*i*_ is the corresponding Plausibility score.

The left hand side of Eq 1 is the logit function, and the right hand side is the linear model including interaction terms between Experience and the two presence variables. For each of the parameters *β*_*j*_, *j* = 0,…,5 we give independent N(0,1000) prior distributions—i.e., Normal with mean 0 and variance 1000.

We use the JAGS system [33] to simulate the posterior distributions of the *p*_*i*_ and *β*_*j*_. Of interest are whether the *β*_*j*_ may be inferred to be positive or negative. For example, if *β*_5_>0 it would mean (other things being equal) that Plausibility contributes to the tendency of GPs to prescribe.

Table 1 shows information about the coefficient estimates, and the corresponding posterior distributions are shown in S5–S10 Figs. Although the posterior probabilities are not overwhelming there is a consistent pattern suggesting that both Place Illusion and Plausibility influence the propensity to prescribe the antibiotics, and differentially for the Trainees and GPs. For example, it we take the Trainees (GP = 0) then the only contributors are the intercept, which is positive (meaning greater likelihood of prescription) but there is some evidence of increasing *p* with Place Illusion but decreasing with Plausibility. However, for the GPs (examining the interaction terms) the signs of these coefficients invert.

**Table 1:** Estimates from the Posterior Distributions of the Parameters for the binary logistic model for Prescription.

Parameter Coefficients	Mean ± SD	Probability	95% highest density interval
β_0_ Intercept	24.3 ± 14.4	P(β_0_ > 0) = 0.99	0.5 to 54.0
β_1_ Coeff. of GP	-19.2 ± 15.0	P(β_1_ < 0) = 0.92	-50.6 to 7.1
β_2_ Coeff. of PI	3.9 ± 4.6	P(β_2_ > 0) = 0.82	-3.9 to 14.1
β_3_ Coeff. of GP×PI	-4.3 ± 4.6	P(β_3_ < 0) = 0.85	-14.4 to 3.7
β_4_ Coeff. of Psi	-6.9 ± 4.3	P(β_4_ < 0) = 0.98	-16.2 to 0.2
β_5_ Coeff. of GP×Psi	6.2 ± 4.4	P(β_5_ > 0) = 0.96	-1.2 to 15.8

**Fig 4:**
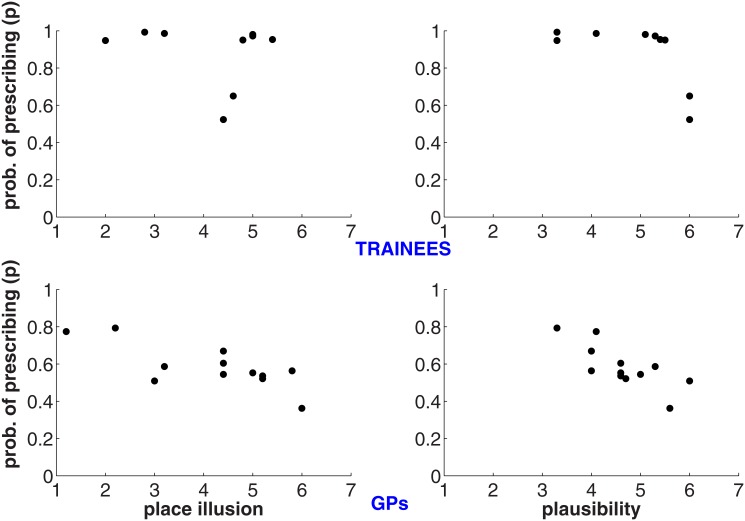
Scatter plots of estimated probability of prescription by place illusion and plausibility.

This can be clearly seen in Fig 4. This shows on the vertical axis the mean probability of prescription (*p* in Eq 1) by Place Illusion and Plausibility. For the Trainees the majority of the probabilities are high, and there are two outliers that account for the results shown in Table 1. For the GPs the relationship is clear—the greater the degree of presence, the less the estimated probability of prescribing the antibiotics.

### Observations and Comments

S1 Video (high resolution version available online: http://youtu.be/KhcnvdKbHrM) shows some typical examples of the scenario and doctor reactions. The experimenters observed that all the doctors were engaged with the scenario, seemed to take it seriously, and from their tone of voice and gestures seemed to react as if the situation were a real one.

As part of the post experiment questionnaire, the participants were asked to write some comments. Some, for example, found the experience stressful:

“I thought this scenario was a good representation of a common issue in General Practice. I felt like it was quite easy to engage with the headset and feel like I was part of the consultation rather than watching a simulation play out. I could see the situation developing and the issue it was examining and I became a little uncomfortable when the time came to say that no antibiotics were necessary.”

“The responses to my questions felt realistic and it felt as if I was in the consultation room. Sometimes it felt stressful as I felt the characters were putting me under pressure to make a decision to prescribe, especially at the end when time was cut short.”

Several participants commented that IVR would be useful in GP trainings:

“Really useful for testing how you respond to difficult situations, would be useful to try again using different consultation techniques. A realistic dilemma—frequently see patients demanding a particular treatment who won't take no for an answer.”

“Powerful tool for this type of dilemma situation e.g. prescribing antibiotics.”

However, a few participants pointed out that there were some aspects of the scenario that required improvement:

“Thought the people were slightly robotic without facial expression which lessened impact about what they were saying but actual content of dialogue very realistic and produced appropriate realistic response from me as if I was in a consulting room.”

“The time delay and visual quality of the patients made me remember it was not real but once I got into the scenario despite these it did feel real. It was challenging as non verbal communication was lost and the words I said were all that mattered it seemed, not even how I said them.”

## Discussion

Doctors are have a high workload and are subject to many interruptions during the course of a working day [34] and it has been estimated that 20% are subject to burnout [35]. Our first concern was whether members of such a pressed profession would afford time to take part in a study that required them to travel to another location from their normal place of work (18 out of the 21 participants had to travel to our lab from relatively distant buildings), don unusual equipment and indulge in ‘virtual reality’ which typically in the mind of the public is associated with video games.

The fact that we were able to recruit 21 doctors and trainee doctors in a very limited amount of time to take part in and complete the study itself points to the positive prospect of using this approach in routine medical training. The observed engagement of the doctors in the process, and their different behavior according to their experience adds weight to this conclusion.

Although the results must be regarded as preliminary, they are based on a change from strongly non-informative prior probabilities that nevertheless result in posterior probabilities that point to a pattern: more experienced doctors are less likely to prescribe antibiotics in the face of strong patient demand, and their probability of prescribing decreases the greater the illusion of presence they have. From the post-experiment comments of the doctors the most important improvements required are in the realm of Plausibility—the role of the attendant medical student was disconcerting for some, pauses in the responses of the virtual characters, their relative lack of facial expression, and so on, all diminished the quality of the experience. These are all factors that can be improved by appropriate scenario design and programming. The doctors generally agreed, however, that the type of scenario shown was in itself useful.

It should be noted that in measuring the doctors and trainee doctors’ responses to the situation, we framed our result around a binary variable: this was simply measured by the participants’ answer to a post-experimental question “Did you prescribe the antibiotics?” where they could choose “yes” or “no”. However, during the experiment it was observed that some participants have chosen to give “delayed prescription”. In future experiments, we will include “delayed prescription” as an outcome. It should also be noted that due to our small sample size, we could not draw any conclusive relationship between other independent variables (e.g., participants’ personality, Burnout Inventory, Professional Identification Scale) and their diagnosis. In future, we hope to conduct large-scale experiments that could further investigate these dimensions.

Technically, there is also space for improvement: in our current version once an utterance for a certain virtual character was triggered, the experimenter would have to wait until the end of the utterance before we could trigger another utterance. This is common practice in real-time character animation in order to avoid jerky and unnatural animations. However, this could also be problematic when for instance the participants decide to interrupt the virtual character in the middle of their sentences, or when the experimenter accidentally triggers an utterance by mistake. Therefore, we are currently working on developing an updated version with a “stop button” that stops the current utterance without producing jerky animation. In future, we should also implement facial expressions on the virtual characters.

The main strength of this study is that it is highly interdisciplinary with researchers from Virtual Reality (computing), neuroscience, law and ethics, and medicine. During our project, not only researchers from humanities learned the power of Virtual Reality, but also VR researchers had an opportunity to put our technical skills to produce useful applications. In future, we hope to benefit from the link established during this project and further explore other research questions that are otherwise impossible to tackle without the use of virtual reality.

The idea of virtual patients for training is not new. Benjamin Lok has explored the use of virtual patients in several aspects of medical training over many years: for example, his most recent work at the time of writing investigated empathy training with virtual patients [36]. In a critical literature review, Cook et al. [37] proposed that the ultimate real value of virtual patients will be in the domain of improving clinical reasoning. We propose widening this to the use of virtual patients in training for ethical dilemmas, and also the use of IVR in helping to understand the dynamics involved in patient-doctor social interactions. In this sense this is no different to previous work using IVR that has focused on extreme social situations such as the Milgram obedience study—we can present quite extreme situations knowing that IVR is powerful enough, through the illusions of presence that it affords, that doctors or others in the medical profession will behave realistically in simulated environments.

## Supporting Information

S1 Fig Script flow chart. (TIFF)

S2 Fig Boxplot of personality inventory. (EPS)

S3 Fig Boxplot of professional commitment by Experience. (EPS)

S4 Fig Boxplot of burnout by Experience. (EPS)

S5 Fig Posterior distribution of the coefficient of GP. (EPS)

S6 Fig Posterior distribution of the coefficient of GP×PI. (EPS)

S7 Fig Posterior distribution of the coefficient of GP×Psi. (EPS)

S8 Fig Posterior distribution of the coefficient of PI. (EPS)

S9 Fig Posterior distribution of the coefficient of Psi. (EPS)

S10 Fig Posterior distribution of the intercept. (EPS)

S1 File The script of the scenario. (PDF)

S2 File Presence questionnaire. (PDF)

S3 File Results of the presence questionnaire and gaze. (PDF)

S4 File Data. (CSV)

S1 Video An overview of the scenario and responses of participants (high resolution version available online http://youtu.be/KhcnvdKbHrM). (M4V)
