# Supplementary figures and images for: The Responses of Medical General Practitioners to Unreasonable Patient Demand for Antibiotics - A Study of Medical Ethics Using Immersive Virtual Reality
Source: PLoS One. 2016 Feb 18;11(2):e0146837. doi: 10.1371/journal.pone.0146837 (PMC4758661; doi:10.1371/journal.pone.0146837)

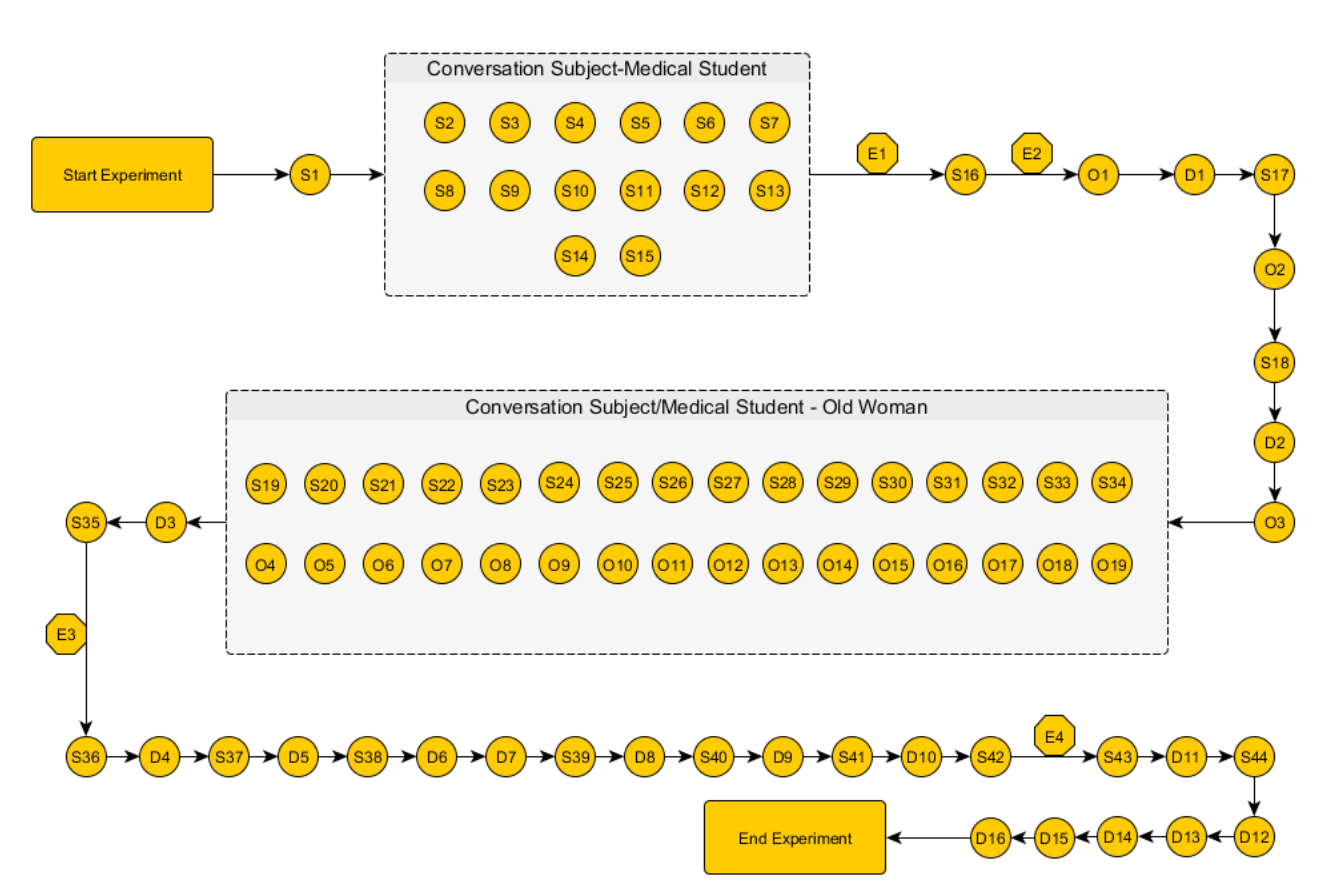

Supplement: S1 Fig — (TIFF) [file pone.0146837.s001.tiff]
