# Supplementary material for: The Responses of Medical General Practitioners to Unreasonable Patient Demand for Antibiotics - A Study of Medical Ethics Using Immersive Virtual Reality
Source: PLoS One. 2016 Feb 18;11(2):e0146837. doi: 10.1371/journal.pone.0146837 (PMC4758661; doi:10.1371/journal.pone.0146837)
Supplement: S1 File — (PDF) [file pone.0146837.s011.pdf]

The Responses of Medical General Practitioners to Unreasonable Patient Demand for Antibiotics - A study of medical ethics using immersive virtual reality

Xueni Pan, Mel Slater, Alejandro Beacco, Xavi Navarro, David Swapp, Joanna Hale, Paul Alexander George Forbes, Catrina Denvir, Antonia F de C Hamilton, Sylvie Delacroix*

*Corresponding Author: s.delacroix@ucl.ac.uk

Supporting Information S1

Script

The participant is seated and enters the virtual reality by putting on the head-mounted display (HMD) and finds him/herself seated at a desk with a screen on top.

There will be an experimental operator who can see and hear everything that is happening, including what the participant is seeing on the HMD, and is able to direct the avatars involved to say or specific things from a menu of choices. This is mainly concerned with timing, but also may play a crucial role in keeping the conversation sensible.

The following Avatars are involved in the scenario: Medical student - S; Old lady - O; Daughter –D

There is about 30s of talk between the virtual medical student. This is mundane talk about nothing of importance, such as the weather.

S: Hello Doctor, I'm David Portilo, a medical student from UCL on attachment to this practice today. How are you?

S: Have you been very busy recently?

S: What do you think of the NHS cuts?

S: If you don't mind me asking, where did you do your medical studies?

S: I see

S: Yes I agree

S: No it doesn't affect me much.

S: Maybe.

S: Let's see what happens.

S: I hope so.

S: Ummm

S: Ah ha [agreeing]

S: My mother is also a doctor, she says things have changed a lot.

S: I am enjoying it yes.

S: It is hard work.

A buzzer sound rings

S: "The last patient has arrived".

S: "Come in".

Door opens, an old lady comes in with her daughter.

O: “Good evening Doctor.”

D: “Hallo Doctor! I am glad that finally we get to see you! We’ve been waiting for an hour! We really need to get this sorted as soon as possible. My mother cannot see very well so I had to drive her here, but we are going away for a couple of days for a wedding in France tonight so we’re in a bit of a rush!”

S: “Hallo, can I just check that the nurse took your temperature and blood pressure outside and looked down your throat and ears - and what seems to be the problem?”

O: “Yes they took my blood pressure, the nurse said it will be on your computer screen,” *cough, cough.* “It was a bit of a wait. But I have to say that it is not as bad as a couple of years ago. There was once I had to wait for 2 hours and the Doctor only saw me for 2 minutes!”

S: Sorry about that we’re always very busy - so what brings you here?

D: “Mum, I think everybody is in a bit of a hurry here.*” Turns to P, smiles and nods* “Sorry”.

O: “Yes, yes…I’m feeling ok but since a couple of days ago I started having a bit of sore throat. This morning, I developed this cough”.

Conversation between Doctor and Old woman (with student’s support if needed)

S: “are you bringing up any phlegm?”

S: “What colour is the sputum?”

S: “Any blood in it?”

S: “Do you have any other symptoms? Any temperature- have you felt shivery or sweaty; short of breath”

S: “Have you been managing to eat ok?”

S: Are you wheezy?

S: Have you been experiencing palpitations?

S: Have you been getting any pains in your chest?

S: Are you able to sleep- how many pillows do you need, what happens when you slide down flat?

S: Are your ankles swelling?

S: Are you having problems keeping your home warm?

S: Is there any damp?

S: Do you generally have any help at home?

S: Are you a smoker?

S: What medication are you on?

S: Are you allergic to any drugs?

O: “I don’t know”

O: “I can’t remember”

O: “All I know is that I’m feeling bad”

O: “Sometimes”

O: “Yes”

O: “No”

O: I feel ok most of the time, but this cough!!

O: I can’t remember what it is called.

O: I’m alone most of the time.

O: Nothing that I know of.

O: Can’t ever sleep well.

O: I listen to the radio at night - helps me to sleep.

O: 1 pillow

O: Oh I never lay like that.

O: Palpitations, what’s that?

O: Am I going to be alright?

Restart of the scripted conversation

D: “My mother had exactly the same thing last year. It started just like this, a couple of days of sore throat, then a cough. But then she became really very ill with a high temperature fever! I had to take a day off work to take her to the hospital! They gave her antibiotics and she immediately got better. So all she needs is some antibiotics this time.”

S: “Let’s check out the blood pressure and throat - it should be on your screen Doctor.”

If the doctor doesn’t do it the student checks on the screen the results.

S: “OK. So what have we got: her throat is a bit red and sore, not too bad. Her ears seem fine, no ear infection. Her chest is completely clear, and her blood pressure is 115/75, pulse 72. Everything seems to be fine. Oh and her temperature is…36.5. No fever.”

D: “Not yet! I bet she will have a fever tomorrow! It always starts like this. Doctor, she needs antibiotics now. There is no point waiting for the fever.”

S: “Well doctor, from the evidence we have so far I don’t think…”

D: “Why do we need to wait until things get worse? Her immune system is not very strong, as you can see. There is no point let her suffering through this. All we need is some antibiotics.”

S: “Doctor I think that we should hold off antibiotics for now- this is just a mild viral illness at this stage”

D: “I am sure it is NOT! Many people in my office have gone down with a nasty cold – and they all had antibiotics. It is the same bug that got her. ”

D: “Even if IT IS JUST A VIRUS, isn’t it better that we give her some antibiotics to help her immune system?”

S: “No, it doesn’t always help and could cause her other problems. Research has shown that antibiotics do no good in many situations, and very often have very unpleasant side effects! ”

D: “So what do you suggest?”

S: “She needs to drink plenty of hot drinks. A light diet, steam inhalations and keep mobile. If she gets any worse we can always review her – I’ll prescribe some paracetamol and menthol and eucalyptus steam inhalations

D: “Doctor, the problem is that we are going away tonight to France for a couple of days. My mother cannot come in on her own because she cannot drive!”

S: “Then you can take her in when you come back? Or find someone else to take her in?”

D: “No, we are going away, and we cannot just leave without sorting this out for my mother. What if she gets really ill while we are away? We are her only family here. Nobody else will be able to take her in!”

S: “Doctor I will give them the emergency number for her to contact us. Is there a neighbour who can help?”

[*Landline rings* “Ding....”]

S: “Hello…yes, ok I will ask. Doctor, reception says that the next patient needs to be seen urgently, right away!”

D: Please can you give us the antiobiotic prescription now, and we will only use it if things get worse.

S [*speak to the phone*]: “Yes it is OK.”

D: “Doctor, maybe you can give us the prescription, but we will only collect it if my mum gets worse?”

If the doctor says no, the daughter gets really upset

D (*upset*): This is so unfair. It's going to ruin our holiday. I'm going to take this up with the local health authority; I think you are unfairly denying medicine to my mother….

D: My mother worked and paid national health insurance all her life. Now look at what's happening!

D: They say on the news that old people are just invisible, not treated with respect by the NHS.

D: I suppose that this is all government cuts to the NHS, but that's just an excuse, you could give the antibiotics if you wanted to.

There could be a few extra phrases like this.

Here it should be allowed to end, since the whole point is to see finally what the doctor does.
