# Supplementary material for: The Responses of Medical General Practitioners to Unreasonable Patient Demand for Antibiotics - A Study of Medical Ethics Using Immersive Virtual Reality
Source: PLoS One. 2016 Feb 18;11(2):e0146837. doi: 10.1371/journal.pone.0146837 (PMC4758661; doi:10.1371/journal.pone.0146837)
Supplement: S2 File — (PDF) [file pone.0146837.s012.pdf]

The Responses of Medical General Practitioners to Unreasonable Patient Demand for Antibiotics - A study of medical ethics using immersive virtual reality

Xueni Pan, Mel Slater, Alejandro Beacco, Xavi Navarro, David Swapp, Joanna Hale, Paul Alexander George Forbes, Catrina Denvir, Antonia F de C Hamilton, Sylvie Delacroix*

*Corresponding Author: s.delacroix@ucl.ac.uk

Supporting Information S2

Presence Questionnaire

Please make sure that you answer each question. If you have any queries ask the experimenters who will be nearby.

Please note that in this questionnaire 'VR *laboratory*' refers to the physical space in which the study is taking place, and 'the consultation room' refers to where the medical consultation took place.

1. Please rate *your sense of being in the consultation room*, on the following scale from 1 to 7, where 7 represents your *normal experience of being in a place.*

I had a sense of being there in the consultation room...

at no time 1 2 3 4 5 6 7 almost all the time

2. To what extent were there times during the experience when the *consultation room* was the reality for you?

There were times during the experience when the consultation room was the reality for me...

at no time 1 2 3 4 5 6 7 almost all the time

3. When you think back about your experience, do you think of the *consultation room* more as *images that you saw*, or more as *somewhere that you visited*?

The consultation room seemed to be more like...

images that I saw 1 2 3 4 5 6 7 somewhere I visited

4. During the time of the experience, which was strongest on the whole, your sense of being in the *consultation room*, or of being in the real world of the laboratory?

I had a stronger sense of...

being in the *consultation room* 1 2 3 4 5 6 7 being in the lab

5. During the time of the experience, did you often think to yourself that you were just sitting in a laboratory or did the *consultation room* overwhelm you?

During the experience I was thinking that I was really in the VR laboratory...

most of the time 1 2 3 4 5 6 7 rarely

6. How much did you behave within the *consultation room* as if the situation were real?

I responded as if the situation were real …

not at all 1 2 3 4 5 6 7 very much

7. How much was your emotional response in the interview the same as if it had been real?

My emotional response in the consultation room was the same as if it had been real …

never 1 2 3 4 5 6 7 almost all the time

8. How much were the thoughts you had within the *consultation room* the same as if it had been a real situation?

My thoughts with in the consultation room were the same as if it had been real …

never 1 2 3 4 5 6 7 almost all the time

9. How much were you thinking things like 'I know this isn't real' but then surprisingly finding yourself behaving as if it was real?

In spite of my knowledge that the situation wasn't real I found myself behaving as if it were real…

never 1 2 3 4 5 6 7 almost all the time

10. To what extent were your physical responses within the *consultation room* (e.g., heart rate, blushing, sweating, etc.) the same as if it had been a real situation? (In

this case if in such a real situation you would have had no or few such physical responses and also within the *consultation room* you had no or few physical responses, then your answer should be closer to 7 than to 1).

My physical responses within the consultation room were the same as if it had been real …

never 1 2 3 4 5 6 7 almost all the time

There three others in the experience that you have just had:

11. How much did you behave as if these others were real people?

I behaved as if they were real…

not at all 1 2 3 4 5 6 7 very much

12. How much was your emotional response to the others as if they were real?

My emotional response to them was as if they were real …

not at all 1 2 3 4 5 6 7 very much

13. How much were your thoughts in relation to the others as if they were real?

My thoughts in relation to them were as if they were real …

not at all 1 2 3 4 5 6 7 very much

14. How much did you have physical responses (such as change in heart rate, blushing, sweating, etc.) to the others as if they were real?

My physical responses to them were as if they were real…

not at all 1 2 3 4 5 6 7 very much

15. How much were you thinking things like 'I know these people are not real' but then surprisingly finding yourself behaving as if they were?

In spite of my knowledge that they weren't real I found myself behaving as if they were real…

never 1 2 3 4 5 6 7 almost all the time

Please enter your comments. Things you could consider are:

- Aspects of the experience that made you respond as if it were real.
- Aspects of the experience that led you to make responses that would have been unrealistic if the situation depicted had been occurring in reality.
- Aspects of the situation that suddenly disturbed your experience of being in the interview.
- Aspects of the experience that helped or hindered you achieving your task.
- Aspects of your feelings towards the experience
- Your relationships and views of the three characters involved.
-

Write your answer in the space below:
