# Supplementary material for: The Responses of Medical General Practitioners to Unreasonable Patient Demand for Antibiotics - A Study of Medical Ethics Using Immersive Virtual Reality
Source: PLoS One. 2016 Feb 18;11(2):e0146837. doi: 10.1371/journal.pone.0146837 (PMC4758661; doi:10.1371/journal.pone.0146837)
Supplement: S3 File — (PDF) [file pone.0146837.s013.pdf]

The Responses of Medical General Practitioners to Unreasonable Patient Demand for Antibiotics - A study of medical ethics using immersive virtual reality

Xueni Pan, Mel Slater, Alejandro Beacco, Xavi Navarro, David Swapp, Joanna Hale, Paul Alexander George Forbes, Catrina Denvir, Antonia F de C Hamilton, Sylvie Delacroix*

*Corresponding Author: s.delacroix@ucl.ac.uk

Supporting Information S3

Presence Questionnaire – Results

Place Illusion

Question	Trainee	GP
Please rate *your sense of being in the consultation room*, on the following scale from 1 to 7, where 7 represents your *normal experience of being in a place*	4.3 (1.0)	5.1 (1.7)
To what extent were there times during the experience when the *consultation room* was the reality for you?	3.1 (1.7)	4.5 (1.4)
When you think back about your experience, do you think of the *consultation room* more as *images that you saw*, or more as *somewhere that you visited*?	4.2 (1.6)	3.4 (1.9)
During the time of the experience, which was strongest on the whole, your sense of being in the *consultation room*, or of being in the real world of the laboratory?[a]	4.1 (1.7)	3.9 (1.9)
During the time of the experience, did you often think to yourself that you were just sitting in a laboratory or did the *consultation room* overwhelm you?[a]	4.9 (1.7)	3.8 (2.0)

Questions were on a 1-7 scale where 7 represented greater PI, except for [a] which was reverse coded

Plausibility Illusion

Question	Trainee	GP
How much did you behave within the *consultation room* as if the situation were real?	4.4 (1.4)	4.9 (1.2)
How much was your emotional response in the interview the same as if it had been real?	5.4 (1.4)	4.7 (1.6)
How much were the thoughts you had within the consultation room the same as if it had been a real situation?	5.0 (0.9)	5.5 (1.2)
How much were you thinking things like 'I know this isn't real' but then surprisingly finding yourself behaving as if it was real?	5.1 (1.0)	5.3 (1.4)
To what extent were your physical responses within the consultation room (e.g., heart rate, blushing, sweating, etc.) the same as if it had been a real situation?	4.7 (1.1)	4.6 (1.1)
How much did you behave as if these others were real people?	4.9 (1.6)	4.1 (1.4)
How much was your emotional response to the others as if they were real?	4.9 (1.2)	4.2 (1.2)
How much were your thoughts in relation to the others as if they were real?	4.9 (0.9)	4.4 (1.3)
How much did you have physical responses (such as change in heart rate, blushing, sweating, etc.) to the others as if they were real?	4.7 (1.7)	4.0 (1.0)
How much were you thinking things like 'I know these people are not real' but then surprisingly finding yourself behaving as if they were?	4.3 (1.6)	4.8 (1.5)

Questions were on a 1-7 scale where 7 represented greater Psi.

Gaze Result

Measurement	Trainee	GP
**GazeToStudent**	7% (3%)	7% (1%)
**GazeToMother**	39% (2%)	38% (2%)
**GazeToDaughter**	35% (3%)	36% (3%)
